# Consumers’ Perceptions About and Use of the Internet for Personal Health Records and Health Information Exchange: Analysis of the 2007 Health Information National Trends Survey

**DOI:** 10.2196/jmir.1668

**Published:** 2010-12-18

**Authors:** Kuang-Yi Wen, Gary Kreps, Fang Zhu, Suzanne Miller

**Affiliations:** ^3^Biostatistics and Bioinformatics DepartmentFox Chase Cancer CenterPhiladelphiaUnited States; ^2^Center for Health and Risk CommunicationDepartment of CommunicationGeorge Mason UniversityFairfaxUnited States; ^1^Psychosocial and BioBehavioral MedicineCancer Prevention and ControlFox Chase Cancer CenterPhiladelphiaUnited States

**Keywords:** Internet, personal health records, health information exchange, consumer perceptions and utilization, demography, health care surveys, health communication trend

## Abstract

**Background:**

Personal health records (PHRs) and the sharing of health information through health information exchange (HIE) have been advocated as key new components in the effective delivery of modern health care. It is important to understand consumer attitudes toward utilization of PHRs and HIE to evaluate the public’s willingness to adopt these new health care tools.

**Objective:**

The purpose of this study was to examine consumer attitudes toward PHRs and their health care providers’ use of HIE, as well as to evaluate consumer use of the Internet for tracking PHRs.

**Methods:**

Analysis of data from the 2007 iteration of the Health Information National Trends Study (HINTS, N=7674) was conducted using multivariate logistic regression to identify predictors of consumer (1) appraisal of PHRs, (2) appraisal of health care provider use of HIE, and (3) use of the Internet for tracking PHRs.

**Results:**

: Approximately 86% of US adults rated electronic access to their PHRs as important. However, only 9% of them used the Internet for tracking PHRs. Those who rated electronic access to their PHRs as important were more likely to be Hispanic (odds ratio [OR] = 1.34, 95% confidence interval [CI] 1.04 - 1.72) and Internet users (OR = 1.27, 95% CI = 1.02 - 1.57) and less likely to be age 65 and above (OR = 0.50, 95% CI = 0.38 - 0.67) or individuals whose doctors always ensured their understanding of their health (OR = 0.62, 95% CI = 0.49 – 0.78). Those who rated HIE as important were more likely to be 45 to 54 years of age (OR = 1.46, 95% CI = 1.03 - 2.08), 55 to 64 years of age (OR = 1.83, 95% CI = 1.32 - 2.53), or 65 and above (OR = 1.76, 95% CI = 1.27 - 2.43) and less likely to be women (OR = 0.80, 95% CI = 0.68 - 0.95) or individuals who perceive their health information as not safely guarded by their doctors (OR = 0.53, 95% CI = 0.40 - 0.69). Among Internet users, those who used the Internet to track their PHRs were more likely to be college graduates (OR = 1.84, 95% = 1.32 - 2.59) or to have completed some college courses (OR = 1.46, 95% CI = 1.02 - 2.11), to be Hispanic (OR = 1.92, 95% CI = 1.23 - 2.98), or to be individuals with health care provider access (OR = 1.90, 95% CI = 1.21 - 2.97). Women were less likely to use the Internet for tracking PHRs than men (OR = 0.78, 95% CI = 0.61 - 1.00).

**Conclusions:**

Despite widespread positive appraisal of electronic access to PHRs as important, Internet use for tracking PHRs remains uncommon. To promote PHR adoption, the digital divide associated with the gap in health literacy must be improved, and cultural issues and the doctor-patient relationship need to be studied. Further work also needs to address consumer concerns regarding the security of HIE.

## Introduction

The Institute of Medicine’s 2001 landmark report, Crossing the Quality Chasm, notes that “the advent of the Internet and the World Wide Web has placed us on the threshold of a change that is reshaping virtually all aspects of society, including health care delivery” [1]. The report recommended that “access to care should be provided over the Internet, by telephone, and by other means in addition to in person visits.” In 2005, the Pew Internet and American Life Project survey found that one fifth of Americans who used the Internet reported that the Internet had greatly improved the way that they received information about health care [2]. They also found that 17 million Americans reported that the Internet played a crucial or important role as they helped another person cope with a major illness [2]. According to an analysis of data from the 2003 Health Information National Trends Survey, there were substantial differences between where people preferred to obtain cancer-related information (half preferred to go to health care professionals) and where they actually got this information; consumers actually used the Internet to access health information far more often than getting information from their doctors [3].

Personal health records (PHRs), one of the emerging health informatics technologies, provide powerful and transformative potential for enhancing the delivery of health care. PHRs are electronic applications that consumers can use to enter and exchange their own health data and to access information from their medical records and other resources [4]. Some of these approaches are “tethered” applications to a given institution and largely focus on insuring patient access to data collected in the course of clinical care (eg, PatCIS [5] and PatientSite [6]). Tethered PHRs’ application components continue to expand to include features such as clinical communication capabilities, disease management tools, decision support systems, and patient annotation capabilities, with great potential to advance patient engagement and activate the patient in knowledge-based collaborations with clinicians, resulting in a transformation of the patient-provider relationship and patient-centered care [4,7-10]. “Untethered” PHRs are freestanding repositories into which an individual can document various health behavior observations regarding diet, exercise, smoking, and other lifestyle changes (eg, WebMD, www.webmd.com). It is advocated that many untethered applications will perform these functions superiorly to some tethered PHRs and can be useful supplements to them [11]. Research consistently shows that consumers have growing and significant interest in using PHRs due to employers demanding PHRs to be included in health plans, health care reforms identifying PHRs as solutions, and the market entry of Google and Microsoft into the promotion of PHRs. However, actual utilization of PHRs technologies is still low [12]. More than 60% of people participating in a Deloitte 2008 Survey of Health Consumers reported that they wished they had online access to their medical records [13]. Another public survey administered by the Markle Foundation found that 89% of the survey respondents reported that they would like to review their medical records if they could, and 65% were interested in accessing their own PHRs online [14]. In 2008, a national survey reported that 79% of US consumers agreed that using electronic PHRs could provide significant benefits in managing their health and heath care services [15]. However, only 2.7% of adults have an online PHRs, and 80% of those who have accessed their online PHRs considered it to be valuable [15]. In sum, trends in consumer survey research suggest growing interest in using electronic PHRs but also reflect limited access to them.

Sharing appropriate patient information electronically among different parties and the ability to access medical records online have been cited as high priorities for encouraging health care technology investment and facilitating health care reform [16,17]. Health information exchange (HIE) benefits include providing real-time decision support to clinicians and patients, making critical clinical information available, and reducing unnecessary testing [18,19]. Models also suggest that HIE will have substantial financial benefits [20]. However, issues of patient privacy and data security have often been raised because HIE involves electronically exchanging patient-identified health information across separate entities that might have potential threats to the confidentiality of the information [21,22]. From the patient's perspective, confidentiality is essential to the patient–physician relationship [21,22]. Patients need to be assured that only information crucial to their correct treatment will be disclosed to providers who have bona fide needs for this information. One recent study reported that patients were enthusiastic about HIE, recognizing its capacity to improve the quality and safety of health care despite concerns about the privacy of their health information [23]. Educational materials and thoughtful consenting processes were identified as critical facilitators for patients’ HIE participation and engagement [23]. Another study conducted with primary care patients found that many patients were unwilling to have their personal information distributed other than for the purposes of their clinical care and that they would like to be consulted before their information is released [24]. The high level of interest, as well as concerns, about HIE suggest that more attention should be directed toward achieving a better understanding of consumers’ attitudes and willingness to engage in health information exchange.

The most recent iteration of the Health Information National Trends Survey (HINTS 2007) is an ideal data source for examining the perceptions, prevalence, and user characteristics of Internet applications for PHRs and HIE for US consumers. The HINTS 2007 nationally representative survey contains specific questions with regard to individual attitudes toward using the Internet for personal health information electronic access and exchange. Since HINTS 2007 also includes many demographic and health-related questions, it also allows examination of the association between these primary interest questions and other domains. This study’s specific aims were to identify (1) the sociodemographic and health-related predictors of consumer perceptions about the importance of PHRs and HIE and (2) the prevalence and predictors of use of the Internet for tracking personal health information.

## Methods

### Data Source

This study used data from the publicly accessible 2007 HINTS developed by the National Cancer Institute, a biennial national probability survey of US civilian noninstitutionalized adults. HINTS collects representative data regularly (the 3 iterations were in 2003, 2005, and 2007) to assess the US public’s use of health-related and cancer-related information and perception, knowledge, and behaviors. The primary goal of the HINTS program is to provide updates on health communication usage, trends, and practices across the US population. Information about the HINTS survey conceptual framework and methodology are available elsewhere [25,26]. The 2007 HINTS contains some changes with regard to new survey items (such as items addressing concepts of PHRs and HIE) and a new sampling method to increase response rates and reduce bias [27]. For data collection, 2 formats were used: (1) a random digit dial (RDD) telephone survey with a computer-assisted telephone interview of representative samples of US households with landline telephones (n = 4092) and (2) a pencil-and-paper questionnaire mailed to representative samples of US postal addresses oversampled for minorities (n = 3582). The application of the dual sampling frames was an alternative solution for a recent dramatic decrease in telephone survey response rates and is an effective method currently being employed by other government agencies.

A total of 7674 adults participated in the survey. All participants were asked about their attitudes toward “accessing their medical information electronically” (our question for PHRs importance) and “their health care providers sharing medical information electronically” (our question for HIE importance). Only those 5078 survey participants who had access to the Internet were asked about their “use of the Internet for tracking personal health records” (our question for PHRs use). In the present analysis, we included both final sample weights and replicate weights to obtain population-level estimates with the correct standard errors [27].

### Study Variables

Data for each variable were grouped into categories consistently with other research using HINTS data [28-30].

#### Sociodemographic Variables

Age, gender, education, and race/ethnicity were included in the analysis. Age was categorized into 6 groups: 18 to 24, 25 to 34, 35 to 44, 45 to 54, 55 to 64, and 65 and above. Educational level was categorized as high school degree or less, some college, or college graduate. Race and ethnicity was categorized into 4 categories: non-Hispanic white, non-Hispanic black (African American), Hispanic, and non-Hispanic other. Non-Hispanic other included American Indian, Asian American, Pacific Islander, Native Hawaiian, and Alaskan Native.

#### Health-Related Variables

In all, 3 health-related variables were included. The first was self-identified overall health status, recoded into 2 categories: (1) excellent, very good, or good, and (2) fair or poor. The second was the respondent’s cancer experience with 3 mutually exclusive categories: (1) having had a personal diagnosis of cancer (regardless of whether or not a family member had been diagnosed with cancer), (2) having had a family member diagnosed with cancer, or (3) having had no personal experience or family member with cancer. We also included a health care access variable, indicated by whether the respondent reported having a regular health care provider or not (yes or no response).

#### Internet Access Variable

The question “Do you ever go online to access the Internet or World Wide Web or to send and receive an email?” was used to measure the Internet status of the respondents (yes or no response).

#### Perceived Deficits in Health Care Provider Variable

With respect to individuals’ perceptions of their information comprehended by their providers, we used the question: “How often did they (health care providers) make sure you understood the things you needed to do to take care of your health?” The responses to this question were recoded to 2 categories: (1) always/usually and (2) sometimes/never.

#### Personal Health Information Security Variable

Individuals’ perceptions for the security level of personal health information were measured by the responses to the statement: “In general, I think that the information I give doctors is safely guarded” (agree or disagree response).

#### Outcome Variables: Personal Health Records Perception and Use Variables

Individual importance attitude toward accessing personal electronic medical records was measured by the following question: “How important would it be for you to get your own medical information electronically?” Responses to this question were recorded on a 3-point scale of importance that denoted (1) very important, (2) somewhat important, and (3) not at all important. Use of the Internet for tracking personal health records was assessed by responses to the following question: “In the past 12 months, have you done the following while using the Internet: Kept track of personal health information, such as care received, test results, or upcoming medical appointments?” (Respondents were asked to give a yes or no response.)

#### Outcome Variable: Health Information Exchange Perception Variable

The importance that individuals placed on health information exchange among providers was assessed by the question: “How important is it to you that your health care providers are able to share your medical information with each other electronically?” Responses to this question were recorded on a 3-point scale of importance that denoted (1) very important, (2) somewhat important, and (3) not at all important.

### Data Analysis

Analyses were done using Stata 10.0 (College Station, Texas, USA) package to accommodate the sampling design of HINTS. Any responses of “refused” or “don’t know” were treated as missing values for all analyses. Unknowns were removed from the denominators when calculating percents. We examined 3 outcome variables: (1) How important would it be for you to get your own medical information electronically? (2) How important is it to you that your health care providers are able to share your medical information with each other electronically? (3) In the past 12 months, have you kept track of personal health information, such as care received, test results, or upcoming medical appointments while using the Internet? The data sampling mode effect was tested against all 3 outcome variables. The 2 PHR outcome variables were significantly different between mail and telephone survey samples. To be consistent, mode effect was adjusted for all analyses. All point estimations were adjusted by the final sample weights and the jackknife method was used for the standard error estimations with 100 replicate weights incorporated. Descriptive statistics were calculated for all variables. A separate bivariate analysis was conducted to estimate the proportion in each responsive category of the study variables between Internet users and Internet nonusers. Logistic regression analyses were used to answer the research questions of whether selected sociodemographic and health domain variables predict the individual’s perception for PHRs and HIE, as well as the user behavior of tracking personal health information on the Internet.

## Results

### Characteristics of the Sample Population

In 2007, approximately 69% of the US population reported that they used the Internet. The findings showed that Internet users were more likely to be younger, healthier, non-Hispanic white, with some college education and without a history of cancer diagnosis. (The weighted sample sociodemographic and study variables are summarized between Internet users and Internet nonusers in Table 1). Approximately half of the overall sample perceived the PHRs and HIE as “very important.” Of the Internet users, approximately 15% (772/5078) reported using the Internet to track their personal health information.

**Table 1:** Weighted sample characteristics: proportion of Internet users and Internet nonusers in each category

Characteristic		Internet Users (Total n = 5078, 68.7%)		Internet Nonusers (Total n = 2566, 31.3%)		
		n	Weighted Percent^a^	n	Weighted Percent^a^	*P* Value
**Age**						.001
	18-24	303	16.2	51	6.1	
	25-34	629	19.7	130	13.1	
	35-44	913	22.0	189	13.9	
	45-54	1213	19.9	357	17.6	
	55-64	1137	13.7	464	14.5	
	65+	860	8.4	1344	34.9	
**Gender**						.001
	Male	1934	47.1	1028	51.9	
	Female	3141	52.9	1533	48.1	
**Education**						< .001
	High school or less	1014	27.8	1460	70.7	
	Some colleague	1608	39.8	576	22.2	
	Colleague graduate	2309	32.4	323	7.1	
**Race/ethnicity**						< .001
	Non-Hispanic white	3868	74.5	1561	57.3	
	Hispanic	324	9.4	295	21.1	
	Black/African American	381	9.5	302	15.8	
	Other	283	6.7	140	5.8	
**General health**						< .001
	Excellent, very good, or good	4383	88.4	1736	72.4	
	Fair or poor	545	11.6	622	27.6	
**Cancer experience**						< .001
	No personal experience with cancer	1262	29.5	872	40.0	
	Have family with cancer	3235	64.7	1277	50.6	
	Cancer survivor	581	5.8	417	9.4	
**Have regular health care provider**						< .001
	No	1008	29.1	627	35.1	
	Yes	4035	70.9	1890	64.9	
**How often did they (health care providers) make sure you understood the things you needed to do to take care of your health?**						.77
	Sometimes/never	496	12.9	270	13.3	
	Always/usually	4024	87.1	1885	86.7	
**In general, I think that the information I give doctors is safely guarded.**						.23
	Agree	4370	87.5	2190	89.1	
	Disagree	613	12.5	259	10.9	
**How important would it be for you to get your own medical information electronically**?						< .001
	Very important	2579	46.6	1007	53.6	
	Somewhat important	1767	32.7	762	35.2	
	Not at all important	641	20.7	605	11.2	
**How important is it to you that your healthcare providers are able to share your medical information with each other electronically?**						.04
	Very important	2564	47.2	1304	52.0	
	Somewhat important	1905	42.0	834	37.4	
	Not at all important	492	10.8	243	10.6	
**In the past 12 months, have you done the following while using the Internet: Kept track of personal health information such as care received, test results, or upcoming medical appointments**^b^?				Not applicable	Not applicable	Not applicable
	Yes	772	13.8			
	No	4271	86.2			

^a^ Results were weighted to be representative of the adult population of Internet users residing in the United States. Mail and RDD sample were separately weighted due to different survey mode effect. All analyses were adjusted by survey mode effect.

^b^ The use of the Internet for tracking personal health information was only asked of Internet users.

^c^ *P* values associated with Wald statistics

### Multivariate Analyses

#### Odds of Importance of Accessing Personal Health Records Electronically

Age, racial ethnicity, Internet access, and perceived deficits in information comprehended by health care providers emerged as the significant predictors in the model of perceived importance for accessing personal health records electronically. Individuals aged 65 and above were about half as likely as those aged from 18 to 24 to value the importance of accessing personal health records electronically (odds ratio [OR] = 0.50, 95% confidence interval [CI] = 0.38 - 0.67). Members of the Hispanic population were more likely than non-Hispanic white respondents to value the concept of electronic personal health records (OR = 1.34, 95% CI = 1.04 -1.72). Compared with those who did not have Internet access, Internet users were more likely to positively appraise the importance of accessible electronic personal health records (OR = 1.27, 95% CI = 1.02 - 1.57). Those who reported deficits in information comprehended by their health care provider were more likely than those who reported that their doctors always ensured their understanding of their health to rate accessing personal health records electronically as important (OR = 0.62, 95% CI = 0.49 - 0.78).

#### Odds of Importance of Personal Health Information Exchange Among Health Care Providers

Our analysis showed that age, gender, and perception of personal health data security predicted who was more likely to value the importance of health information exchange among providers. Adults aged 45 to 54, 55 to 64, and 65 and above were more likely than those aged 18 to 24 to rate the use of health information exchange as important, while the age group 55 to 64 reported the highest importance of HIE (OR = 1.83, 95% CI = 1.32 - 2.53). Females were less likely than males to perceive the importance of their health care providers sharing personal health records electronically (OR = 0.80, 95% CI = 0.68 - 0.95). Respondents who perceived their health information was not safely guarded by their doctors were about half as likely to value the importance of health information exchange among providers as those who believed their personal information was secured (OR = 0.53, 95% CI = 0.40 - 0.69).

**Table 2:** Multivariate ordinal logistic regression of predictors of perceived importance for accessing electronic personal health records (n = 7383) and health care provider sharing personal health information electronically (n = 7366)^a^

Characteristic		Odds of Importance of Accessing Personal Health Information Electronically		Odds of Importance for Health Care Providers Sharing Personal Health Information With Each Other Electronically	
		OR (95% CI)	*P* Value^b^	OR (95% CI)	*P* Value^b^
**Age**			< .001		< .001
	18-24	1.00		1.00	
	25-34	0.90 (0.63 - 1.28)	.55	1.03 (0.73 - 1.44)	.88
	35-44	1.03 (0.74 - 1.44)	.84	1.39 (0.97 - 1.98)	.07
	45-54	0.90 (0.66 - 1.22)	.48	1.46 (1.03 - 2.08)	.03
	55-64	0.89 (0.64 - 1.23)	.47	1.83 (1.32 - 2.53)	< .001
	65+	0.50 (0.38 - 0.67)	< .001	1.76 (1.27 - 2.43)	< .001
**Gender**			.45		.01
	Male	1.00		1.00	
	Female	0.94 (0.81 - 1.1)	.45	0.80 (0.68 - 0.95)	.01
**Education**			.86		.66
	High school or less	1.00		1.00	
	Some colleague	1.06 (0.87 - 1.29)	.59	0.91 (0.75 - 1.12)	.37
	Colleague graduate	1.03 (0.82 - 1.29)	.78	0.96 (0.79 - 1.16)	.67
**Race/ethnicity**			.10		.78
	Non-Hispanic white	1.00		1.00	
	Hispanic	1.34 (1.04 - 1.72)	.03	1.04 (0.77 - 1.42)	.80
	Black/African American	1.23 (0.92 - 1.64)	.16	0.89 (0.68 - 1.16)	.38
	Other	1.10 (0.76 - 1.58)	.61	0.89 (0.58 - 1.35)	.58
**General health**			.51		.62
	Excellent, very good, or good	1.00		1.00	
	Fair or poor	1.08 (0.86 - 1.36)	.51	1.07 (0.82 - 1.40)	.62
**Cancer experience**			.43		.46
	No personal experience with cancer	1.00		1.00	
	Have family with cancer	0.90 (0.76 - 1.06)	.21	1.11 (0.92 - 1.34)	.28
	Cancer survivor	0.92 (0.74 - 1.13)	.42	1.15 (0.90 - 1.46)	.25
**Have regular health care provider**			.21		.44
	No	1.00		1.00	
	Yes	0.88 (0.71 - 1.08)	.21	1.10 (0.87 - 1.38)	.44
**Internet access**			.03		.29
	No	1.00		1.00	
	Yes	1.27 (1.02 - 1.57)	.03	0.89 (0.73 - 1.10)	.29
					
**How often did they (health care providers) make sure you understood the things you needed to do to take care of your health?**			< .001		.64
	Sometimes/never	1.00		1.00	
	Always/usually	0.62 (0.49 - 0.78)	< .001	1.05 (0.86 - 1.28)	.64
**In general, I think that the information I give doctors is safely guarded.**			.88		< .001
	Agree	1.00		1.00	
	Disagree	0.98 (0.73 - 1.31)	.88	0.53 (0.40 - 0.69)	< .001

^a^ Results were weighted to be representative of the adult population residing in the United States. All analyses were adjusted by survey mode effect.

^b^ *P* values associated with Wald statistics

#### Odds of Use of Internet for Tracking Personal Health Information

Among Internet users, use of the Internet for tracking personal health information was predicted by gender, race, educational level, and access to a regular health care provider. Females were less likely than males to use the Internet for tracking personal health information (OR = 0.78, 95% CI = 0.61 - 1.00). Those with educational levels more extensive than a high school degree were more likely than those with only a high school degree or less to use the Internet for tracking personal health information (OR = 1.46, 95% CI = 1.02 - 2.11 for those who had some college education compared with those who had high school education or less, and OR = 1.84, 95% CI = 1.32 - 2.58, for college graduates compared with those who had high school education or less). Compared with non-Hispanic white respondents, Hispanic population members were almost twice as likely to use the Internet for tracking personal health information (OR = 1.92, 95% CI = 1.23 - 2.98). Respondents with a regular health care provider were almost twice as likely as those without a regular health care provider to use the Internet for tracking personal health information (OR = 1.90, 95% CI = 1.21 - 2.97).

**Table 3:** Multivariate logistic regression of use of Internet for tracking personal health information among Internet users (n = 5078)^a^

Characteristic		Odds of Using the Internet for Personal Health Information	
		OR (95% CI)	*P*^b^
**Age**			.35
	18-24	1.00	
	25-34	0.91 (0.49 - 1.67)	.75
	35-44	0.84 (0.46 - 1.53)	.56
	45-54	0.91 (0.51 - 1.62)	.74
	55-64	0.96 (0.53 - 1.74)	.88
	65+	1.28 (0.68 - 2.4)	.44
**Gender**			.05
	Male	1.00	
	Female	0.78 (0.61 - 1.00)	.05
**Education**			.002
	High school or less	1.00	
	Some college	1.46 (1.02 - 2.11)	.04
	College graduate	1.84 (1.32 - 2.58)	< .001
**Race/ethnicity**			.04
	Non-Hispanic white	1.00	
	Hispanic	1.92 (1.23 - 2.98)	< .001
	Black/African American	1.21 (0.76 - 1.92)	.43
	Other	1.36 (0.8 - 2.33)	.25
**General health**			.26
	Excellent, very good, or good	1.00	
	Fair or poor	1.25 (0.85 - 1.83)	.26
**Cancer experience**			.40
	No personal experience with cancer	1.00	
	Have family with cancer	0.87 (0.64 - 1.18)	.38
	Cancer survivor	1.04 (0.69 - 1.58)	.85
**Have regular health care provider**			.01
	No	1.00	
	Yes	1.90 (1.21 - 2.97)	.01
**How often did they (health care providers) make sure you understood the things you needed to do to take care of your health?**			.12
	Sometimes/never	1.00	
	Always/usually	0.73 (0.49 - 1.09)	.12
**In general, I think that the information I give doctors is safely guarded.**			.82
	Agree	1.00	
	Disagree	0.95 (0.59 - 1.51)	.82

^a^ Results are weighted to be representative of the adult population of Internet users residing in the United States. All analyses were adjusted by survey mode effect.

^b^ *P* values associated with adjusted Wald statistics

## Discussion

This study examined consumers’ attitudes toward accessing personal health records electronically and their health providers’ health information exchange ability, as well as the prevalence of using the Internet for tracking personal health information to better understand who would value these concepts and who is currently accessing the emerging PHR technologies. The results showed widespread positive appraisal of electronic access to PHRs, which was predicted by younger age, Hispanic ethnicity, Internet access, and perceived deficits in health care provider. The characteristics of older age, male gender, and the belief in personal health information security predicted positive appraisals of health information exchange. Use of the Internet for tracking personal health information was uncommon and was predicted by the following demographic characteristics: male gender, Hispanic ethnicity, higher educational level, and access to a regular health care provider.

### The Importance of Electronic Personal Health Records

#### Age Difference in the Perception of the Importance of PHRs

According to our findings, older adults were less likely than younger adults to value the importance of PHRs. Other studies also found that those aged 65 and over reported placing less value in Internet health information than those younger than age 65 and that those aged 65 and over would be less likely to use the Internet to find health information [31]. As the adoption rate of Internet and broadband use has continued to grow among senior citizens [32] and health problems tend to increase with age, future research needs to examine factors such as improving computer self-efficacy [33] and addressing design issues [34] that promote senior citizens’ value of and intention to use PHRs, which will impact on their chronic care management.

#### Perception of PHRs Among Hispanic Population Members

Members of the Hispanic population more highly valued the concept of electronically accessible medical records than non-Hispanic white respondents in our overall sample. Studies have shown that Hispanic individuals are interested in using the Internet for health information [35], but they are less likely than whites to have access and to use the Internet. Income and education levels do not fully explain the gap in Hispanic individual’s use of the Internet [36]. Cultural factors are more likely to influence perceptions of use of the Internet for health information [37,38]. As technologies evolve, we need to evaluate how cultural factors impact on the design, adoption, and dissemination of PHR applications among different ethnical groups.

#### Internet Access and Perception and Use of PHRs

Almost 46.7% (3586/7674) of the respondents surveyed reported that it was very important to have access to their medical records electronically (32.9% also reported that this was “somewhat important”). In particular, Internet users were more likely than Internet nonusers to report the importance of tracking their personal health information online. However, only 15% of the Internet users had used the Internet for tracking their personal health information in 2007. Our finding is consistent with previous consumer survey research that showed that despite high enthusiasm among consumers for PHRs, the actual uptake of PHRs has been relatively slow [39]. PHR technologies have become increasingly popular among consumers, clinicians, policy makers, and purchasers, and many vendors and health care providers already have the tools available to offer PHRs to their customers [40]. While the uptake of PHRs has been slow, a growing number of patients actively use this emerging technology [41]. We need to continue evaluating barriers to the adoption of PHRs.

#### Perceived Deficits in Information Comprehended by Health Care Providers Positively Associated With Perceived Importance of Personal Health Records

Interestingly, our results showed that respondents who reported a lack of attention from their health care providers to ensure their understanding and comprehension of their personal health were more likely to value the importance of accessing their medical records electronically. This suggests the possibility of these consumers perceiving PHRs as a compensating tool for gathering their personal health information they are not receiving from their doctors. In a related finding, Zickmund et al [42] in studying a diabetes patient portal with online information, laboratory results, and secured messaging, found that patients’ interest in the portal was linked to dissatisfaction with their doctor-patient relationship. Individuals may be more willing to reach out for alternative modes of computer-mediated information and communication if they have a dissatisfying relationship with their providers. To fully understand the potential of PHRs for providing consumers with relevant health information, further studies are needed to determine changes in both patients’ and providers’ attitudes regarding the use of PHRs and the impact of PHR use on the doctor-patient relationship.

### The Importance of Health Information Exchange

#### Age Difference in Perceptions of the Importance of HIE

Respondents in the youngest group studied (aged 18 to 24) in the sample were less likely to value the importance of HIE compared with respondents who were aged 35 and above. The discrepancy in perceptions of HIE importance between younger and older generations might reflect the difference in experience interacting within the health care system. Since younger consumers are generally healthier than older consumers, younger consumers tend to have fewer concerns about their personal health histories and have less frequent interactions within the health care system. The meaning of HIE is likely to be much different for younger consumers than for members of older generations who potentially have more experience with illness and health care providers due to the aging process.

#### Gender Difference in Perceptions of the Importance of HIE

Men were more likely than women in this survey to positively appraise the importance of HIE in 2007. This finding may suggest greater comfort with using information technologies and interacting with health care providers among men, although previous research has suggested that women generally have greater concern for health issues and actively seek health information more often than men [30]. The general importance afforded to HIE by men, however, suggests an opportunity to expand HIE programs and services for men.

#### Consumer Concerns About HIE Security

It is argued that building privacy and security protection into HIE systems will bolster the public trust and confidence that are critical to the rapid adoption of HIE and to the realization of its benefits [43]. Not surprisingly, our analysis revealed that consumers’ attitudes toward HIE were significantly influenced by the perceived level of security of their personal health information managed by their providers. The security and privacy issue has been recognized as a significant barrier to electronic HIE, which requires the implementation and establishment of national privacy principles, trusted network design characteristics, and oversight and accountability mechanisms [43]. To fully engage consumers in health information technology innovations, it may be wise to use health literacy principles to develop simple but clear patient consent and educational materials explaining privacy and security precautions [44]. Recently, the Consumer Education and Engagement Collaborative was formed to develop a series of coordinated, state-specific projects to educate consumers about privacy and security to make them fully aware of current information-sharing practices and policy discussions [45]. Future initiatives must be designed to build awareness and trust for new health information technologies within society to facilitate HIE adoption and to influence its use in health care. We also need to carefully design policies relating to patient consent without placing an undue burden on health care professionals [46].

### The Use of the Internet for Tracking Personal Health Information

#### Higher Use of PHRs Among Hispanic Population Members

It is interesting to note in our findings that members of the Hispanic population who had Internet access were more likely to use the Internet for tracking personal health information than non-Hispanic white respondents after adjusting for age, educational level, and so on. Although Hispanic individuals and members of other minority groups are substantially less likely to have a home computer and use the Internet than non-Hispanic whites [47], there is strong evidence that eHealth systems will be used extensively with a positive impact on underserved minority populations who have access to such technology [48].

#### Health Literacy Impacts on the Adoption and Use of PHRs

Our finding in this study suggests that PHR use is generally associated with higher educational levels among all Internet users. Kaiser Permanente’s PHR study also reported that their PHR registration was associated with higher educational levels [49]. As the adoption rate of Internet and broadband has continued to grow among those with less than a high school education [50], patients with limited health literacy may not be able to easily understand the information available on PHRs, thus limiting the benefit from such health communication tools [51]. To provide optimal benefits to the patient, PHRs must present data, information, terminology, and accompanying tools in ways that allow the patient to understand and to act on the information learned [4]. Thus, PHR development should focus on meeting patients’ health information preferences and capabilities. Integrating patient-centered testing throughout PHR development is essential to ensure the readability and usability of PHRs. Early assessment, testing, and prompt initiation of training to address literacy issues is critical to ensure successful PHR adoption [52]. Computer skills and technological literacy is another related yet critical concern that needs to be addressed. Whether individuals are technology savvy enough to update personal records and interact with health information systems may also impede adoption of PHRs.

#### Gender Difference in the Prevalence of Use of PHRs

To our surprise, our findings revealed that men who were online were more likely to use the Internet for tracking personal health information compared with women who were online. In contrast to our findings, past Internet research indicated that women were more likely than men to search for health information [30], use online patient provider communication [53], and use online support groups or health-based chat rooms [54]. Similar findings of gender differences in online health searching were also reported by the Pew Internet and American Life Project [55]. However, other studies also showed that men tend to use the Internet for instructional support while women tend to use the Internet to seek emotional support [56].

#### Health Care Provider Access Associated With PHR Use

The association between having access to a health provider and PHR use was also observed, suggesting that Internet users with a regular provider were more likely to use PHRs. The result is consistent with findings that individuals with a regular primary care provider are more likely to use eHealth services than those who do not have a regular primary care provider [57]. It is likely that HINTS respondents with a regular health care provider were more likely to be health conscious, and health consciousness has been shown to influence preventive health care behaviors [58] and online health information-seeking behaviors [59].

#### Cancer and Health Status With Relation to PHRs

We found that neither cancer history nor general health status were associated with PHR use. Previous eHealth studies have shown that Internet users with more medical needs tend to use eHealth services more frequently [53]. However, HINTS survey respondents who reported that they had been diagnosed with cancer could currently be in remission and may not have been actively coping with the disease. Therefore, we might not accurately distinguish healthy versus less healthy respondents in our analysis [28]. Also, eHealth interventions have been identified as most valuable for individuals with chronic conditions [60,61]. Pew project research has suggested that individuals with chronic disease who have Internet access are more likely to search for health information online than those without a chronic condition [62]. Access to appropriate health information is a key support function for cancer patients. PHRs and related eHealth services provide an effective mechanism of patient access, which is consistent with the increasing preference of cancer patients for personalized information according to their medical records [63]. Future research is needed to further investigate the potential of PHR use for cancer patients.

### Limitations

The HINTS survey question asked only about use of the Internet for tracking personal health information, such as care received, test results, or upcoming medical appointments. However, people may use the Internet for many health purposes (such as documenting drug prescriptions with applications like GoogleHealth or to view insurance claim data with the use of subscriber portals provided by insurance companies), which is consistent with the concept of PHRs but may not have been identified by survey respondents as “use the Internet for tracking personal health information.” Thus, we might underestimate the prevalence of Internet use for PHRs. HINTS data were based on self-report with potential bias due to social desirability, which may challenge the generalizability of the results. In addition, the HINTS survey does not allow for further examinations of barriers to the use of PHRs or the perceived benefits for those who use these tools. Many PHRs also include unique functionality that allows patients to send secured messages to their providers. Research examining online doctor-patient communication using the HINTS data has been reported elsewhere [53].

Due to item wording, we can only discuss our results with regard to HIE as a way for “health care providers to share medical information with each other electronically” and cannot characterize the HIE mechanisms in different formats (ie, consumers who exchange their personal information with providers) that could potentially affect our findings concerning consumers’ perceived value of HIE. We were limited to examination of the use of PHRs rather than HIE with the survey instrument. People may be engaging in HIE through their health care providers in ways that were not reported in the survey. Further examination of consumer participation in HIE would provide new insights into the use of the Internet for exchanging personal health data.

To improve the delivery of health care we need to continue to assess consumers’ and health care providers’ perspectives on barriers and benefits related to using the Internet for PHRs and HIE as health information technologies evolve rapidly as part of clinical practice.

### Conclusions

Personal health records and health information exchange are critical tools for reengineering our health care system. Significant future research is needed to understand the adoption of PHRs and HIE as integrated tools that improve patient-centered care and care coordination and to identify the barriers and impact of their use on patients, providers, organizations, and health care systems across clinical, financial, and behavioral outcomes.

Although current dissemination of PHRs and HIE into clinical care is limited, the advocacy of stakeholder groups, demand from patients, and strong push for health care reform are likely to accelerate the adoption of these important technologies. However, just making the technologies accessible and available is not sufficient. In 2009, the Health Information Technology for Economic and Clinical Health (HITECH) Act authorized incentive payments through the Center of Medicare and Medicaid Services to clinicians and hospitals when they demonstrate meaningful use of certified electronic health records privately and securely. The proposed definition of meaningful use includes ways not only for health care providers to store and retrieve patient medical information but also for patients and families to gain access to their medical records and thus engage more fully and collaboratively in their care [64,65.] Health care agencies and research communities need to ensure the readability and usability of PHR tools to meet the needs of diverse populations with varying levels of health and computer literacy [40,66]. Supporting the patient’s transitions between care settings or personnel is also part of the meaningful use objectives. Attention must be given to critical issues inherent to the use of HIE, including security, privacy, and confidentiality. Clear information and policies about data management and transaction and security and privacy issues need to be rigorously defined and disseminated to sustain consumer trust.

In sum, we need to continue addressing policies and establish architectures at both state and federal levels that support the development and implementation of PHRs and HIE and that account for both consumer and health care provider needs and preferences. Critical issues with regard to system usability and interoperability, health literacy and cultural issues, data security, and health care costs need to be addressed for maximizing the wide dissemination of PHRs and HIE.

## Abbreviations

HIE: health information exchange
HINTS: Health Information National Trends Study
HITECH: Health Information Technology for Economic and Clinical Health
PHRs: personal health records
RDD: random digit dial
